# Detection of common biogenic amines in fermented sausage produced in Turkey

**DOI:** 10.1016/j.dib.2018.08.089

**Published:** 2018-08-29

**Authors:** Kamil Ekici, Abdullah Khalid Omer

**Affiliations:** a University of Yȕzȕncȕ Yıl, Veterinary College, Department of Food Hygiene and Technology, Van, Turkey; b Sulaimani Veterinary Directorate, Veterinary Quarantine, Bashmakh International Gate, Sulaimani, Iraq

## Abstract

The application of HPLC method to detect the BA concentration in fermented sausage was successful and proved that HPLC system and method served its purposes. Biogenic amines are generated in foods as a result of free amino acid decarboxylation by bacterial enzymes. Biogenic amines accumulations are unwanted in all foods and beverages because if consumed at high concentrations, they may induce foodborne intoxications. Histamine, putrescine, cadaverine, tyramine, tryptamine, beta-phenylethylamine, spermine and spermidine are considered to be the most important biogenic amines occurring in foods. The determination of biogenic amines in foods is of great interest due to their potential toxicity and can be used as indicators for food quality markers.

**Specifications Table**

**Table:** 

Subject area	*Agricultural and Biological Science*
More specific subject area	*Food Hygiene and Safety*
Type of data	*Table, text file*
How data was acquired	*HPLC (Agilent Technologies, Germany), vacuum degasser, DAD detector, and a computer including the Agilent package program.*
Data format	*Analyzed*
Experimental factors	*Common biogenic amine determination. To avoid contamination Sausage were sliced with a clean stainless steel knife, and transferred into falcon plastic tubes then homogenized and immediately prepared for analysis.*
Experimental features	*Obtained data were analyzed with HPLC method*
Data source location	*Van, Turkey*
Data accessibility	

**Value of the data**

• Data presented here provide confirm HPLC method for the separation, quantification and determination of biogenic amine in fermented sausage.
• The results showed that the use of HPLC technique was relatively simple to carry out, selective, accurate, sensitive, repeatable, reproducible and robust for the quantification, separation, and determination of biogenic amine.
• Outcomes have also essential and significant data especially for doctors and dieticians and they must have enough information on biogenic amines in foods in order to improve and reduce the potential toxicity of amines.

## 1 Data

Naturally biogenic amines present in humans, animals, plants, and microbes. The existence of the biogenic amine of food components potential public health significance because of physiological, psychological, and toxicological impacts [1]. Table 1 shows the physiological and pharmacological effects of biogenic amines. The presence of high quantities of these amines in fermented sausages should be advised as a consequence of a poor hygienic quality of raw materials. Differentiation in the biogenic amines concentrations of sausages could be due to the hygienic quality of raw material, manufacturing practices, the specific bacteria, ripening period and the type of culture.

**Table 1:** Biogenic amines in foods and their physiological and pharmacological effects [2], [3].

Amine	Precursors	Physiological effect	Pharmacological effect
Histamine	Histidine	Neurotransmitter, local hormone, gastric acid secretion, cell growth and differentiation, regulation of circadian rhythm, body temperature, food intake, learning and memory, immune response, allergic reactions.	Liberates adrenaline and noradrenaline. Excites the smooth muscles of the uterus, the intestine, and the respiratory tract. Stimulates both sensory and motor neurons. Control gastric secretions.
Tyramine	Tyrosine	Neurotransmitter, peripheral vasoconstriction, increase cardiac output, increase respiration, elevate blood glucose, release of norepinephrine.	Peripheral vasoconstriction. Increases the cardiac output, respiration and blood sugar level. Causes lacrimation and salivation. Releases noradrenaline from the sympathetic nervous system. Causes migraine.
Putrescine	Ornithine	Regulation of gene expression maturation of intestine, cell growth and differentiation.	Hypotension. Bradycardia. Lockjaw. Paresis of the extremities. Potentiate the toxicity of other amines.

The detection of biogenic amines in foodstuffs is of great interest because of their potential toxicity and can be used as indicators for nourishment quality markers [4], [5]. Various analytical techniques for the detection of biogenic amines in foodstuffs have been identified. HPLC is commonly recommended for the separation and quantification of biogenic amines among all techniques.

## 2 Experimental design, materials and methods

### 2.1 Materials

Turkish fermented sausages (which is composed of beef meat, sheep tail fat, water buffalo, clean dry garlic, sugar, salt, some spices (for example, red pepper, black pepper, cinnamon and cumin), sodium nitrate, sodium nitrite, ascorbic acid, olive oil, antioxidants and antimicrobials) were purchased from retail shops in the Van city of Turkey, and investigated for biogenic amine contents.

### 2.2 Biogenic amine analysis

Biogenic amines analyses were done by using HPLC method as described by Eerola et al. [6].
