# Supplementary material for: Detection of common biogenic amines in fermented sausage produced in Turkey
Source: Data Brief. 2018 Aug 29;20:1360–2. doi: 10.1016/j.dib.2018.08.089 (PMC6146452; doi:10.1016/j.dib.2018.08.089)
Supplement: Supplementary file 1 — Supporting information [file mmc1.docx]

Conflict of interest

All the authors confirm as No conflict of interest.
